# Detection of *Balamuthia mandrillaris *DNA by real-time PCR targeting the RNase P gene

**DOI:** 10.1186/1471-2180-8-210

**Published:** 2008-12-03

**Authors:** Albrecht F Kiderlen, Elke Radam, Astrid Lewin

**Affiliations:** 1 Robert Koch Institute, Cellular Immunology Unit P22, Nordufer 20, 13353 Berlin, Germany

## Abstract

**Background:**

The free-living amoeba *Balamuthia mandrillaris *may cause fatal encephalitis both in immunocompromised and in – apparently – immunocompetent humans and other mammalian species. Rapid, specific, sensitive, and reliable detection requiring little pathogen-specific expertise is an absolute prerequisite for a successful therapy and a welcome tool for both experimental and epidemiological research.

**Results:**

A real-time polymerase chain reaction assay using TaqMan^® ^probes (real-time PCR) was established specifically targeting the RNase P gene of *B. mandrillaris *amoebae. The assay detected at least 2 (down to 0.5) genomes of *B. mandrillaris *grown in axenic culture. It did not react with DNA from closely related *Acanthamoeba *(3 species), nor with DNA from *Toxoplasma gondii*, *Leishmania major*, *Pneumocystis murina*, *Mycobacterium bovis *(BCG), human brain, various mouse organs, or from human and murine cell lines. The assay efficiently detected *B. mandrillaris *DNA in spiked cell cultures, spiked murine organ homogenates, *B. mandrillaris*-infected mice, and CNS tissue-DNA preparations from 2 patients with proven cerebral balamuthiasis. This novel primer set was successfully combined with a published set that targets the *B. mandrillaris *18S rRNA gene in a duplex real-time PCR assay to ensure maximum specificity and as a precaution against false negative results.

**Conclusion:**

A real-time PCR assay for *B. mandrillaris *amoebae is presented, that is highly specific, sensitive, and reliable and thus suited both for diagnosis and for research.

## Background

Balamuthiasis is a disease of humans and a variety of mammalian species caused by the free-living amoeba *Balamuthia mandrillaris *[1,2]. Its most important clinical manifestation is *Balamuthia *amoebic encephalitis (BAE), also described as granulomatous amebic encephalitis (GAE), in both immunocompromized hosts and in individuals apparently without immunological deficits [3,4]. *B. mandrillaris *is exquisitely encephalotropic and cytopathic [5], causing extensive brain tissue damage [6]. With worldwide around 150 identified cases to date [7], BAE is rare, however, with only 3 documented survivors [8,9], exceedingly lethal. Due to lack of practical experience, amoebic encephalitis is often mistaken for a brain tumour, viral or bacterial encephalitis, tuberculoma, or neurocysticercosis [3]. There exist no characteristic clinical symptoms, nor laboratory, or radiological findings diagnostic of BAE [10]. The high percentage of fatal BAE cases is also due to the high frequency of misdiagnosis and subsequent false, possibly even exacerbating, treatment strategies. At the moment, a proper diagnosis is possible only at autopsy, which is seldom done in most countries. Conversely, BAE is most likely severely under-diagnosed and thus reviewed by some authors as a genuine emerging [11] or at least potentially threatening [12] parasitic infection.

*B. mandrillaris *amoebae have recently been detected in soils both in the close vicinity of and distant from a BAE case [13,14]. Inhalation of contaminated dust or wounding contact with contaminated soils are the likely sources of infection. While infections of the skin and lungs may prevail for months, the infection of the central nervous system (CNS) can result in death in a matter of days [15]. As patients generally present first with neurological symptoms indicating that the CNS is already affected, rapid and dependable diagnosis is an absolutely prerequisite for any successful therapy.

Until recently, diagnosis of BAE relied on microscopical analysis of brain tissue (biopsy and necropsy), with indirect immunofluorescence (IIF) microscopy as the favoured detection method [2,16,17]. Apart from specific antiserum, this requires appreciable expertise, and a tissue sample of sufficient quality. PCR-based methods have the advantage of requiring much less pathogen-specific experience and can therefore be easily added to the diagnostic panel of any laboratory equipped with the basic tools and know-how. In addition, they are potentially more sensitive and specific, and can analyse larger sample volumes. Finally, they do not necessarily rely on intact pathogens and are more likely to detect an infection, i.e. pathogen-specific nucleic acid, in the blood or CSF, enabling less invasive diagnostic procedures.

PCR-based methods are being increasingly used for detecting known pathogenic amoebae [18-20]. To date, *B. mandrillaris *PCRs generally target ribosomal RNA (rRNA) gene sequences [17,21-24]. Even when quantification is not necessary, real-time PCR should be given preference over conventional PCR whenever possible, as the omission of post-amplification handling leads to quicker results and reduces the risk of amplicon contamination [24-26]. To identify false-positive results due to cross-reactivity and to ensure that novel variants or mutants in the target gene do not lead to false-negative results, the identification of microorganisms by PCR should involve the detection of at least 2 independent areas of the pathogen's genome in parallel. This becomes increasingly important with the microorganisms pathogenicity, when misdiagnosis bears grave consequences [27,28]. Accordingly, a real-time PCR assay was established specifically targeting the RNase P gene [29] of *B. mandrillaris *and tested in normal and spiked cell cultures, organ samples and patient material. Furthermore, this new assay was compared to a recently published real-time PCR targeting an 18S rRNA gene sequence of *B. mandrillaris *[24], and a duplex assay combining both was performed for optimal specificity and reliability.

## Results and discussion

The aim of this communication is to introduce a real-time PCR-based assay for detecting infections with *B. mandrillaris *amoebae with maximum specificity, sensitivity, and reliability. For this, a partial RNase P gene sequence, published in GenBank, was found containing a region(nucleotide 251 to nucleotide 328) specific for *B. mandrillaris *as demonstrated by basic local alignment search tool (BLAST) analysis. PCR primers and a TaqMan probe were designed as described in Materials and Methods. The enzyme RNase P is responsible for generating the mature 5'-end of tRNA by a single endonucleolytic cleavage of their precursors. It is an essential, ubiquitous enzyme present in all cells and cellular compartments that synthesize tRNA: bacterial cells, *Archaea*, eukaryotic nuclei, mitochondria, and chloroplasts. All known RNase P enzymes are ribonucleoproteins containing an RNA subunit essential for catalysis [29]. Eukaryotic RNase P RNA contains a conserved core structure as well as regions of highly variable length and structure [30].

The ability of the designed RNase P primer set to detect *B. mandrillaris *DNA was evaluated with trophozoites (ca. 10% cysts) from axenic culture. In more than 30 experiments, less than 2 *B. mandrillaris *amoebae equivalents were dependably detected, in individual experiments down to 0.5. The RNase P primer set did not react with ≥ 500 pg DNA from 3 different *Acanthamoeba *species, other protozoa or mycobacteria, nor did it react with mammalian tissues or cell lines. It did, however, detect *B. mandrillaris *DNA in murine tissues spiked with *B. mandrillaris *amoebae (summarized in Table 1). The genus *Acanthamoeba *is closely related to *Balamuthia *[31] and 25-fold polyploid [32]. Assuming that a single *B. mandrillaris *cell contains 25 copies of the RNase P gene, a sensitivity limit of 2 amoebae or 50 gene copies seems reasonable.

**Table 1:** *Balamuthia mandrillaris *RNase P genomic DNA detected by real-time PCR – specificity and detection limits

	**DNA** **[pg] ^a)^**	**Amoebae** **equivalents ^b)^**	**CT ^c)^**
*B. mandrillaris *(axenic culture)	5 – 50	< 2	35 – 38
*Acanthamoeba hatchetti *2HH (axenic culture)	≥ 500	neg	≥ 39
*A. lenticulata *72/2 (axenic culture)	≥ 500	neg	≥ 39
*A. castellanii *1BU (axenic culture)	≥ 500	neg	≥ 39
*Toxoplasma gondii *(partially purified)	≥ 500	neg	≥ 39
*Leishmania major *(axenic culture)	≥ 500	neg	≥ 39
*Pneumocystis murina *(partially purified)	≥ 500	neg	≥ 39
*Mycobacterium bovis *(BCG)	≥ 500	neg	≥ 39
Murine macrophage RAW 264.7 cells (TIB 71)	≥ 500	neg	≥ 39
Human neuroblastoma Kelly cells (ACC 355)	≥ 500	neg	≥ 39
Brain tissue (mouse, ca. 15 mm^3^)	≥ 500	neg	≥ 39
Brain tissue spiked with 1–10 *B.m.*	n.d.	1 – 10	33 – 38
Lung, liver, spinal chord (mouse, ca. 15 mm^3^)	> 500	neg	≥ 39
Lung tissue spiked with 10 *B.m.*	n.d.	10	35–36

^a) ^Minimal amounts of total DNA giving positive results (CT < 39). ^b) ^Minimal numbers of amoebae (calculated as amoeba DNA equivalents per PCR tube) giving positive results; neg = CT ≥ 39 for at least 500 ng total DNA. ^c) ^Range of CT values measured; neg = CT ≥ 39 or no DNA detected; n.d. = not done.

**Figure 1:**
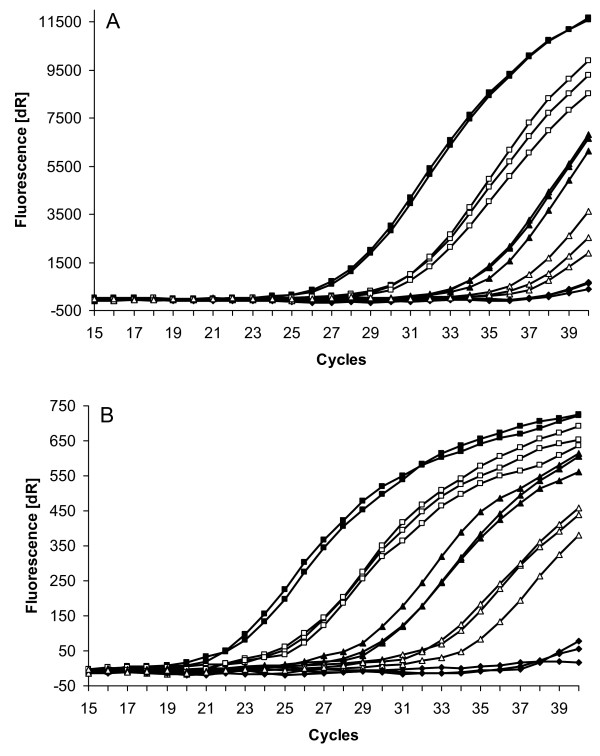
**Detection of *B. mandrillaris *RNase P (A)and 18S rRNA gene (B) DNA by duplex real-time PCR**. Amplification curves of murine brain tissue (ca. 15 mm^3^) alone (closed diamonds) or spiked with 500 (closed squares), 50 (open squares), 5 (closed triangles), and 0,5 (open triangles) axenically cultured *B. mandrillaris *(calculated as amoeba DNA equivalents per PCR tube). Results from 3 separate experiments.

To establish assay sensitivity in the presence of host tissue, pieces of murine brain tissue were spiked with graded numbers of *B. mandrillaris *from axenic cultures (containing up to 10% cysts) and RNase P and 18S rRNA genes DNA detected in parallel by duplex real-time PCR. As show in Fig 1A, down to 0.5 amoeba equivalents could be detected when targeting both RNase P and 18S rRNA genomic DNA.

Our results targeting the RNase P gene revealed a similar sensitivity as those published by Qvarnstrom and colleagues for the 18S rRNA gene in a triplex real-time PCR [24]. In direct comparison (duplex real-time PCR), however, the 18S rRNA gene primer set (Fig. 1B) regularly was more sensitive by 2.5 to 4 cycles than the RNase P gene primer set (Fig. 1A). The 18S rRNA gene is 24-fold repetitive in *A. castellanii *[33]. Assuming *B. mandrillaris *to be 25-fold polyploid, one cell would contain 600 copies of the 18S rRNA gene. A 10-fold difference in gene copies normally results in a difference in sensitivity by 2–3 cycles in the real-time PCR. Thus the assumed 24-fold surplus of 18S rRNA genes per cell might well explain the somewhat lower sensitivity of the RNase P gene primer set.

These promising in vitro results encouraged testing stored material from experimentally infected animals. For example, a brain necropsy specimen that had been kept frozen at -70°C for over 3 years from an intranasally infected mouse that had shown neurological symptoms of BAE before death, and whose brains had been found to be infected with *B. mandrillaris *amoeba by IIF microscopy, also gave positive results by real-time PCR (Table 2). All specimens from noninfected mouse brains were negative.

**Table 2:** *B. mandrillaris *RNase P genomic DNA in infected brain tissues detected by real-time PCR

	**DNA** **[pg] ^c)^**	**Amoebae** **equivalents ^d)^**	**CT ^e)^**
Murine brain tissue from non-infected mice	> 500	neg	≥ 39
Murine brain tissue from *B.m.*-infected mice ^a)^	n.d.	n.d.	30

Human brain tissue from non-infected patients	n.d.	neg	≥ 39
Human brain tissue spiked with 50 *B.m.*	n.d.	50	29
Human brain tissue from BAE-patient 1 ^b) ^(1:100)	n.d.	n.d.	30
Human brain tissue from BAE-patient 2 ^b) ^(1:10)	n.d.	n.d.	35

^a) ^A highly symptomatic CD4^+ ^T cell-deficient mouse 14 days post intranasal infection with *B. mandrillaris *amoebae. ^b) ^DNA extracted from 2 fresh BAE-patient brain biopsy samples ([23] Table 1: cases 4 and 5). ^c) ^Minimal amounts of total DNA giving positive results (CT < 39). ^d) ^Minimal numbers of amoebae (calculated as amoeba DNA equivalents per PCR tube) giving positive results; neg = CT ≥ 39 for at least 500 ng total DNA. ^e) ^Range of CT values measured; neg = CT ≥ 39 or no DNA detected; n.d. = not done.

Next, the real-time PCR assay was applied to fresh brain biopsy specimens from a tumor patient without or with added *B. mandrillaris *amoebae. Human brain tissue had no effect on the assay, as it neither produced false negative results nor inhibited the detection of *B. mandrillaris *DNA (Table 2). Finally, DNA from brain biopsies of 2 BAE patients [23] (Table 1: cases 4 and 5) were tested positive for *B. mandrillaris *RNase P genes (Table 2). Highly similar results were achieved with a second primer set batch, this time using Cy5/BHQ-2 for dye/quencher (data not shown). No differences in assay sensitivity were detected between *B. mandrillaris *DNA from human or baboon BAE cases, supporting the concept of a single genotype for *B. mandrillaris *[34].

## Conclusion

This TaqMan real-time PCR assay using a primer set designed to target a *B. mandrillaris*-specific region of the RNase P gene, detects *B. mandrillaris *genomes in amoeba cultures, spiked tissues, and infected brain tissue with high specificity and sensitivity. In direct comparison with a published primer set that targets the 18S rRNA gene of *B. mandrillaris*, this assay is somewhat less sensitive, which can be explained by the different expected genome numbers per cell of the respective target genes. Both primer sets were successfully combined in a duplex real-time PCR assay to ensure maximum specificity and as a precaution against false negative results.

## Methods

### Mice

Female C57BL/6 wild-type (WT) and immunodeficient C57BL/6 *rag1*^-/- ^(RAG) mice aged 8–12 weeks and bred at the Central Animal Facility (ZVZ), Federal Institute for Risk Assessment (BfR), Berlin, were kept in individually ventilated cages (Bio A.S., Ehret, Emmendingen, Germany) in the animal facility of the Robert Koch Institute. Housing materials, food and drink (both given *ad libitum*) were sterilized by autoclaving or irradiating before use, and all handling was performed in a class-II safety cabinet.

### Cells, microorganisms, and clinical specimens

*B. mandrillaris *(CDC-V039; ATCC 50209) was grown in Chang's special medium as described [5]. *Acanthamoeba hatchetti *(strain 2HH; ATCC PRA-113), *A. lenticulata *(strain 72/2; ATCC 50705), and *A. castellanii *(strain 1BU; ATCC PRA-113) were kindly provided by Julia Walochnik, Clinical Institute of Hygiene, University of Vienna, Austria, and grown axenically in PYG medium as described [35]. Murine macrophage-like RAW 264.7 cells (ATCC TIB 71) and human neuroblastoma Kelly cells (DSMZ ACC 355) were grown in humidified, CO_2_-enriched (5%) normal atmosphere at 37°C in R10 consisting of RPMI 1640 medium (Gibco BRL-Life Technologies, Paisley, UK) supplemented with 10% (v/v) fetal calf serum (FCS; Boehringer Mannheim, Mannheim, Germany), 10 mM Na-pyruvate (Sigma-Aldrich, Steinheim, Germany), 100 IU/ml penicillin, and 100 μg/ml streptomycin (both Gibco). *Leishmania major *LV39 strain was grown at 25°C in R10. *Pneumocystis murina *organisms, originally kindly provided by E. Dei-Cas, INSERM Unit 42, Villeneuve d'Ascq, France, were contained in partially purified lung homogenates from experimentally infected RAG mice. *Mycobacterium bovis *BCG (Copenhagen) was grown at 37°C in Middlebrook medium containing 10% ADC enrichment (Becton Dickinson). PCR-tested *Toxoplasma gondii *(RH-strain) DNA and total DNA from the ileum of *T. gondii*-infected mice was kindly provided by O. Liesenfeld and U. Lohmann, Department of Microbiology and Hygiene, Charité University Medicine Berlin, Germany.

*Balamuthia *mitochondrial 16S rRNA gene-positive total DNA extracted from brain tissue of two reported BAE patients ([23] Table 1: cases 4 and 5) was kindly provided by S. Yagi, California Department of Health Services, Richmond, USA.

### Extraction of DNA

DNA from normal human brain tissue, mouse tissues, cell lines, and amoebae was isolated by a modification of the UNSET procedure [36] as described by Walochnik and coworkers [19]. Briefly, tissue samples of approximately 2.5 × 2.5 × 2.5 mm or the indicated number of cells were solubilized at room temperature (RT) in 500 μl UNSET lysis buffer (8 M urea, 0.15 M NaCl, 2% SDS, 0.001 M EDTA, 0.1 M Tris/HCl; pH 7.5) by repeated (1–2 min) pipetting using a 1 ml disposable pipette tip until a quasi homogenous liquid was achieved. This was overlaid with 250 μl phenol and 250 μl chloroform (PC), and shaken gently (rocking table) for 3–5 h at RT. DNA was extracted by multiple PC-extraction followed by a single chloroform-extraction and precipitated in a 2.5-fold volume of 100% ethanol and a 0.1-fold volume of 3 M Na-acetate (pH 4.8 – 5.3) over night at -20°C, and then centrifuged for 30 min at 12,000 × g and 4°C. The supernatant was aspirated and the pellet washed with 2 ml of 70% ethanol, vortexed, and centrifuged for 5 min at 12,000 × g and 4°C. Alcohol was removed by aspiration and the open tubes placed in a laminar air-flow cabinet for ca. 10 min to evaporate the remaining alcohol before adding ca. 30 μl ddH_2_O (depending on the expected quantity of DNA). The DNA concentration was determined in a UV/Vis spectrophotometer (ND-1000, NanoDrop Technologies, Wilmington, USA). DNA from BCG was extracted as described by Somerville et al. [37].

### Infection of mice with *B. mandrillaris *amoebae

Intranasal infection with *B. mandrillaris *(CDC-V039 was performed as described [38]. In short, 5 × 10^3 ^amoebae (~10% cysts) in 7.5 μl saline were injected into each nostril of CD4-depleted (experimental details will be published elsewhere) anesthetized mice using an Eppendorf pipette fitted with an ultra-micro tip (Eppendorf-Netheler-Hinz, Hamburg, Germany). Around 14 days later, when the mice had become severely ill, they were first anesthetized, then exsanguinated, and individual organs or ~15 mm^3 ^blocks thereof shock-frozen in liquid nitrogen and stored at -70°C for over 3 years.

### Real-time PCR assay

*B. mandrillaris *sequences published in GenBank include rRNA gene sequences and a partial RNase P gene sequence. A nucleotide-nucleotide BLAST analysis with the partial RNase P gene sequence (accession AF440362) revealed the region from nucleotide 251 to nucleotide 328 to be specific for *B. mandrillaris*. Particularly, this region was specific for *B. mandrillaris *also with respect to the *Acanthamoeba *sequences available in the nucleotide databases. The sequence of this specific DNA region was exported to the Primer Express software (Applied Biosystems, Foster City, CA, USA) for the design of the PCR primers and a TaqMan probe. The selected primers RNase P/FW (5'-GGC AGG TTC CGA GGA GAC A-3') and RNase P/RV (5'-GTG GCC TTG TGT ATT GAA CTT AAC ATT-3') amplify a 82 bp region and were used together with the FAM/TAMRA-labelled TaqMan probe RNase P/Probe (5'-Fam-TGG AAC CAT ACC TTG GGT GAC ACG ATG-Tamra-3').

For comparison and design of a duplex real-time PCR assay, the primers and probe recently published by Qvarnstrom et al. [24] were used, whereby the probe was labelled with HEX/BHQ: BalaF1451/FW (5'-TAA CCT GCT AAA TAG TCA TGC CAA T-3'), BalaR1621/RV (5'-CAA ACT TCC CTC GGC TAA TCA-3', and BalaP1582/Probe (5'-Hex-AG TAC TTC TAC CAA TCC AAC CGC CA-Bhq1-3').

The PCR reactions were performed using the ABsolute™ QPCR Mix from ABgene (Epsom, UK) according to the manufacturer's instructions in a total volume of 50 μl. The reaction mix contained 0.4 μM of each primer and 0.1 μM of the probe. PCR runs were performed in the Mx3005P real-time thermocycler from Stratagene (La Jolla, CA, USA) with one hold at 95°C for 15 min and 40 cycles at 95°C for 15 sec (denaturation) followed by 60°C for 1 min (annealing/elongation). The fluorescence data were collected at the end of the annealing/elongation step. Data were analyzed and plotted with the MxPro software from Stratagene.

## Abbreviations

BAE: *Balamuthia *amoebic encephalitis; BCG: Bacille Calmette-Guérin; BLAST: basic local alignment research tool; CD: cluster of differentiation; CNS: central nervous system; CT: threshold cycle; IIF: indirect immunofluorescence; PCR: polymerase chain reaction.

## Authors' contributions

AFK designed the project and wrote the manuscript. ER performed real-time PCR and analyzed the experimental data. AL designed the primer sets and analyzed the data.
